# Spontaneous whole genome duplication renders mouse embryonic fibroblasts resistant to reprogramming

**DOI:** 10.1186/s13578-026-01558-3

**Published:** 2026-03-26

**Authors:** Wei Li, Lingyu Zhong, Pengli Li, Ziwei Zhai, Runxia Lin, Minjing Ke, Yixin Fan, Yu Liu, Yu Fu, Yue Qin, Chengchen Zhao, Bo Wang, Junqi Kuang, Duanqing Pei

**Affiliations:** 1 Guangzhou Institutes of Biomedicine and Health, Chinese Academy of Sciences, Guangzhou, 510530 China; 2 University of Chinese Academy of Sciences, Beijing, 100049 China; 3 Guangdong Provincial Key Laboratory of Stem Cell and Regenerative Medicine, Guangzhou Institutes of Biomedicine and Health, Chinese Academy of Sciences, Guangzhou, 510530 China; 4 Laboratory of Cell Fate Control, School of Life Sciences, Westlake University, Hangzhou, China; 5 Centre for Regenerative Medicine and Health, Hong Kong Institute of Science & Innovation, Chinese Academy of Sciences, Hong Kong, China; 6 Westlake Laboratory of Life Sciences and Biomedicine, Hangzhou, China; 7 Zhejiang Key Laboratory of Biomedical Intelligent Computing Technology, Hangzhou, China; 8 Zhejiang University of Science and Technology School of Information and Electronic Engineering, Hangzhou, China

**Keywords:** Whole-genome duplication (WGD), Tetraploid cells, Cell fate control, Cell fate transition, Somatic cell reprogramming, p38, p53

## Abstract

Whole-genome duplication (WGD) is thought to be quite rare, but has important implications ranging from emergences of new species to tumorigenesis. The burden of doubled genome for a cell must be traumatic and remains poorly understood. Here we report a spontaneous and robust WGD system amicable to detailed molecular analysis. We first show that mouse embryonic fibroblasts (MEFs) undergo spontaneous WGD *in vitro*. The resulting binucleated cells or BNCs become resistant to iPSC reprogramming. We further show that pretreating MEFs with p38 inhibitor SB203580 suppresses WGD and enhances reprogramming. Mechanistically, we demonstrate the WGD activates p53 in BNCs to act as the primary barrier to reprogramming, as its depletion rescues iPSC formation in tetraploid cells. These findings provide a convenient WGD experimental system and our preliminary analysis reveals p38 coordinates WGD progression and p53 regulates subsequent cell fate determination in genome-doubled cells.

**Supplementary Information:**

The online version contains supplementary material available at 10.1186/s13578-026-01558-3.

## Introduction

Whole-genome duplication (WGD) has been recognised as an important mechanism to generate new species during evolution and also give rise to more than 30% of cancer incidences [1–6]. The unscheduled WGD, resulting from cytokinesis failure, endoreplication or slippage, is recognized as a driver of DNA damage and genomic instability in tumors and a facilitator of adaptive plasticity during speciation [3, 7–13]. Moreover, WGD has also been reported to change 3D genome architecture and drive oncogenic loss of chromatin segregation [14, 15]. Little is known about tetraploid cells in terms of cell fate control and the associated regulatory pathways.

Reprogramming of somatic cells (e.g., MEFs) by Yamanaka factors (Oct4/Sox2/Klf4/c-Myc) provides a well-established model for studying cell fate control [16, 17]. We and others have previously identified a series of critical biological events during this process, such as mesenchymal-to-epithelial transition (MET), as well as reprogramming barriers, including AP1 family of transcription factors such as cJUN, epigenetic modifications (e.g., H3K9me3) and DNA methylation [18–23]. Although these were identified in diploid cells, little is known whether tetraploid cells can be reprogrammed to pluripotency.

Here we report that primary MEFs undergo spontaneous WGD in culture and the resulting tetraploid cells are resistant to reprogramming. Our results provide a model to study WGD experimentally and reveal p38 and p53 in regulating WGD and tetraploid reprogramming, respectively.

## Results

### MEFs undergo spontaneous WGD in vitro

**Fig. 1:**
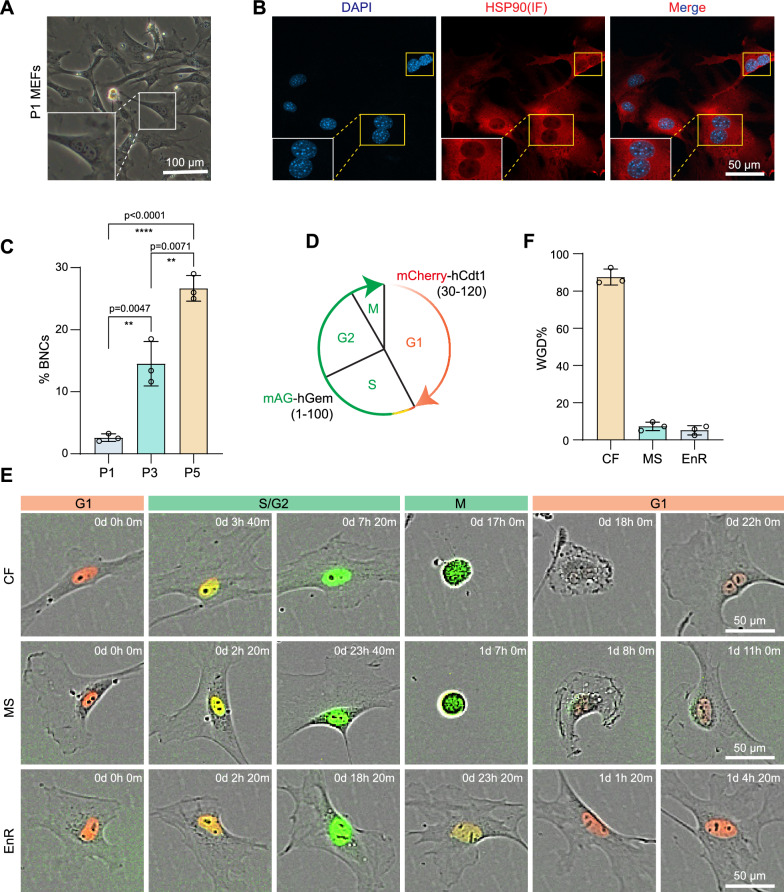
MEFs spontaneously undergo WGD. **A**, Representative images of the BNCs in vitro in MEF culture. The white box highlighted the BNCs. Scale bars, 50 μm. **B**, Representative images of the HSP90 immunofluorescence, mainly located at cytoplasm, which can distinguish between MNCs and BNCs. Scale bars, 50 μm. **C**, Histogram shows the proportion of BNCs in MEFs of at different passages. Data are mean ± s.d., two-tailed, unpaired *t*-test, *N* = 3 strains. **p < 0.01; ****p < 0.0001. **D**, Schematic illustration shows the cell cycle reporter system FUCCI [24]. G1 phase is indicated by mCherry-Cdt1 (30–120) and the S/G2/M phase is marked by mAG-Geminin (1–110). **E**, Time-lapse imaging of MEFs tracked the process of cell division (see also video S1–S3). The cell cycle is indicated by FUCCI. CF, cytokinesis failure; MS, mitotic slippage; EnR, endoreplication. Scale bars, 50 μm. **F**, Histogram shows the proportions of WGD in distinct type. total 150 cells were counted in 3 biological replicates

When we culture primary mouse embryonic fibroblasts (MEFs), we often encounter binucleated cells or BNCs (Fig. 1A). By DAPI staining, we can observe BNCs accumulate steadily during passaging (Fig. 1B–C). By passage 5, BNCs account for close to 30% of the cell population (Fig. 1C). Based on the morphological data in Fig. 1A and B, the BNCs should have undergone whole-genome duplication or WGD. To confirm their DNA contents qualitatively, we measure DNA content by FACS and show that indeed we can detect 2, 4 and 8 N cells within the populations as we analysed for passages 1 (P1) and 3 (P3) (Fig. S1A). Notably, the 8 N cells account for 1 and 5.41% for P1 and P3 respectively (Fig. S1A–B), consistent with the morphological data scored in Fig. 1A. As primary MEFs at P1 also have a low, but strikingly consistent 8 N cells (Fig. S1A–B). Additionally, we did not observe a notable rise in senescence-associated β‑galactosidase staining and ROS levels after WGD (Fig. S2A–D). We believe that WGD is a spontaneous event intrinsic to the cells and the culturing conditions.

We further confirmed this finding with Karyotyping, which showed that while 20 MEFs at P0 are diploid cells, but 10 out of 19 MEFs at P3 are tetraploids (Fig. S1C). To further characterize WGD in MEFs, we took advantage of the FUCCI system [24] to monitor its cell cycle progression (Fig. 1D). Surprisingly, we can identify three types of WGD in the live-cell imaging of MEFs; among all observed WGD events in MEFs, the majority resulted from spontaneous cytokinesis failure (CF, > 80%), and the minor portion were mitotic slippage (MS, < 10%) and endoreplication (EnR, < 10%) respectively (Fig. 1E–F, Video S1–S3). Moreover, roughly 30% of the binucleated cells divided to form mononuclear tetraploid cells (Fig. S3A). These results further confirm spontaneous WGD and suggest that WGD is primarily due to CF.

We noticed an apparent inconsistency between BNCs scored under the microscope, 4N/8N cells by FACS, and Karyotyping, demonstrating WGDs at various ratios from ~ 15%, ~ 5% and > 50% (10/19). As these three different methods are fundamentally different as each detects nuclei, DNA contents and chromosomes, these three different parameters are consistent that WGD takes place with MEFs at a dynamic fashion which can be accurately described with gross cell morphology, DNA contents and karyotypes. As such, we believe that these parameters are in fact consistent in reflecting the intrinsic dynamics of cells undergoing WGD.

### Tetraploid MEFs are refractory to reprogramming

**Fig. 2:**
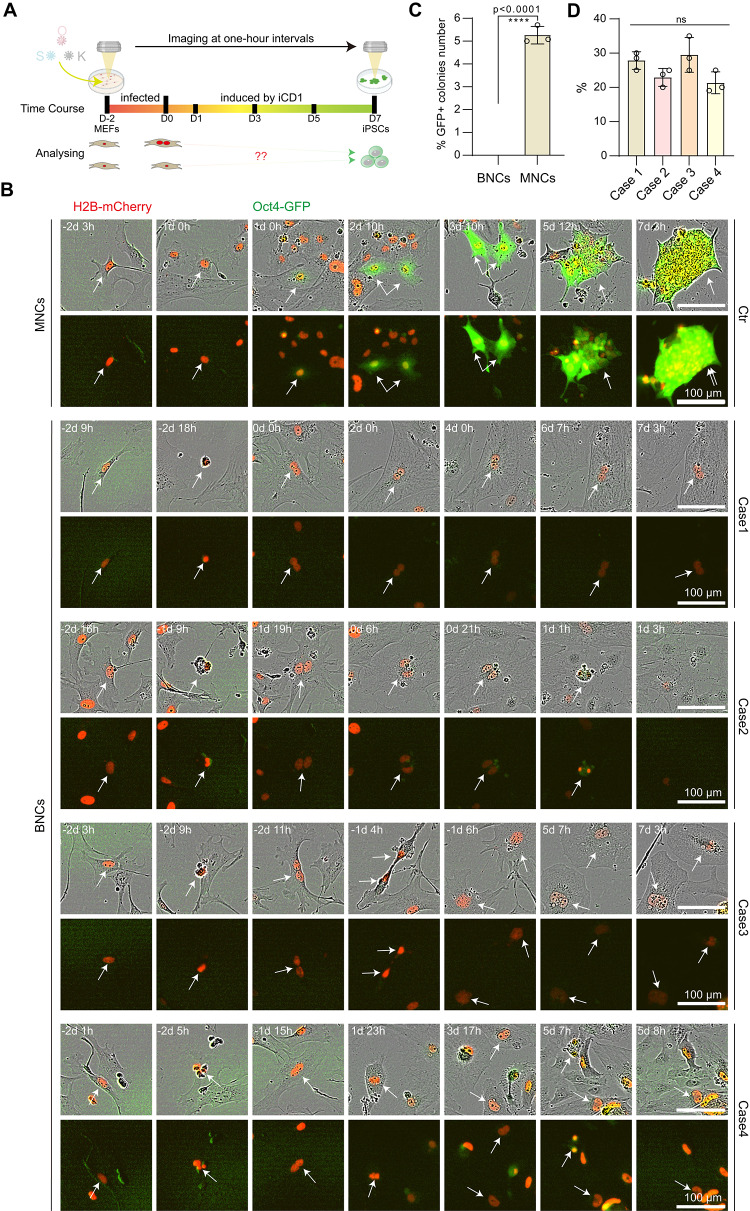
Binucleated MEFs are refractory to reprogramming. **A**, Schematic illustration depicts the live-cell imaging procedure during the reprogramming of MEFs to iPSCs, along with the analytical strategy for binucleated MEF reprogramming. **B**, Time course live-cell images of mononucleated and binucleated MEFs during reprogramming (see also video S4–S8). H2B-mCherry marks nucleus. Scale bars, 100 μm. **C**, Histogram presents the proportion of GFP + colonies number between binucleated and mononucleated MEFs. Total 250 binucleated or MNCs were counted in 3 biological replicates and each biological replicate involved 8 views. Data are mean ± s.d., two-tailed, unpaired *t*-test. ****p < 0.0001. **D**, Histogram shows the proportion of different cases in (C) during binucleated MEFs reprogramming. ns, not significant

WGD is often considered very rare, thus, quite difficult to analyse at the molecular level. Our findings on MEF WGD seem to provide an unique opportunity as a model to this field. We ask whether WGD impacts one well investigated properties of MEFs, i.e., the ability of MEFs to undergo reprogramming towards pluripotency efficiently. To this end, we utilized previously described iCD1-SKO (SOX2, KLF4 and OCT4) reprogramming system with MEFs harboring the OG2 (Oct4-GFP) reporter. This system is characterized by high efficiency, stability and chemically defined culture medium [25–27]. During the iCD1-SKO reprogramming process, live-cell imaging was performed from the initiation of viral infection until 7 days post iCD1 induction to monitor GFP-positive colony formation. To discriminate between mononucleated cells or MNCs and BNCs following cell division, we ectopically expressed the H2B-mCherry fusion protein to fluorescently label nuclei. Given that exogenous retroviral vectors exclusively infect the proliferative cells, our investigation specifically examined whether BNCs resulting from cytokinesis failure during the viral infection window (D-2 to D0) maintained reprogramming competence, as assessed by GFP-positive colony formation (Fig. 2A). In other words, at the viral infection stage, the MEFs under analysis were undergoing cell division, thereby enabling their susceptibility to infection by exogenous viral particles. Our long-term live-cell tracking suggests that approximately 5% of P3 MNCs formed GFP + colonies, whereas BNCs completely lost their reprogramming capacity (Fig. 2B–C). Interestingly, we show that BNCs apparently adopt four distinct fate outcomes or cases during reprogramming: two static (binucleation maintenance or death) and two dynamic fates (successful cytokinesis producing either quiescent MNCs or apoptotic progeny) (Fig. 2B, Video S4–8). Moreover, these four cell fate patterns seem to be similarly distributed (Fig. 2D), suggesting that reprogramming has no preference among these 4 outcomes. These results demonstrate that MEFs subjected to WGD are refractory to reprogramming, suggesting that WGD erects novel barriers for cell fate transition during reprogramming.

To further test this idea, we induced BNCs from MEFs with dihydrocytochalasin B (DCB) [28], an inhibitor of actin polymerization that prevents cytokinesis (Fig. S4A–B). Consistently with results from spontaneous WGD, DCB-treated MEFs exhibited substantially impaired capability to form GFP + colonies (Fig. S4C–D). Together, these findings demonstrate that WGD generates novel barriers for cell fate transition during reprogramming.

### p38i prevents MEF WGD to facilitate reprogramming

**Fig. 3:**
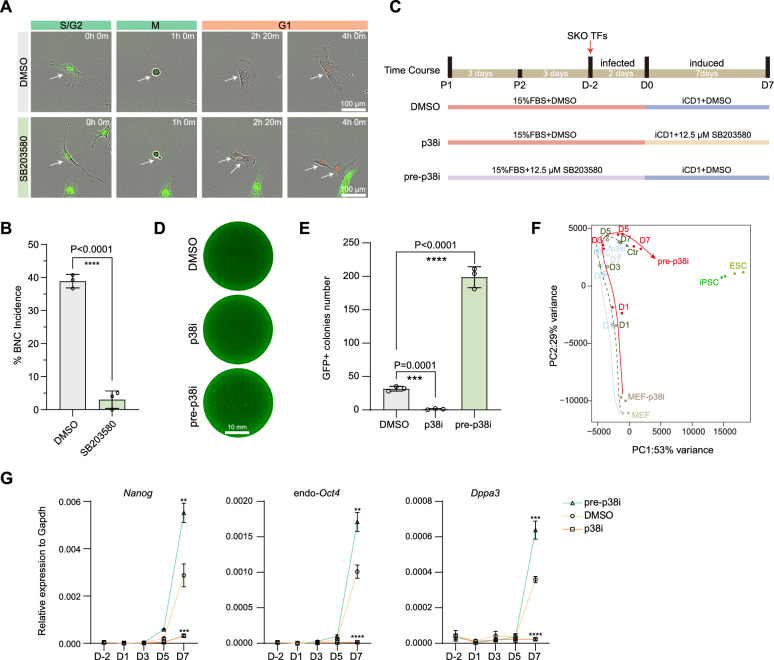
p38i pretreatment impedes WGD and enhances reprogramming. **A**, Time-lapse images of P2 MEFs with or without 12.5 μM SB203580 (Selleck, S1076) (see also Video S9–S10). Scale bars, 100 μm. The arrows indicated the tracked cells and their daughter cells. **B**, The proportion of BNCs incidence is quantified via live-cell FUCCI in P2 MEFs with or without 12.5 μM203580. Cells in mitotic period were counted from randomly 3 views. Data are mean ± s.d., two-tailed, unpaired *t*-test, ****p < 0.0001. **C**, Schematic of SB203580 treatment in different phases: MEFs were cultured with SB203580 before iCD1 induction, designated as pre-p38i; SB203580 was added during the iCD1 induction window, referred to as p38i, along with the corresponding DMSO control. **D**, Representative images of GFP + colonies at D7 in different period treated with SB203580. Scale bar, 10 mm. **E**, Histogram shows the GFP + colonies number at D7 in indicated group. Data are mean ± s.d., two-tailed, unpaired *t* test; *n* = 3 independent experiments, ***p < 0.001; ****p < 0.0001. **F**, PCA analysis of all samples across various timepoints using RNA-seq data. G, RT–qPCR analysis of the expression of representative pluripotent genes. Data are mean ± s.d., two-tailed, unpaired* t* test; *n* = 3 biological replicates. **p < 0.01; ***p < 0.001; ****p < 0.0001

We next wish to test a hypothesis that inhibiting WGD in MEFs can enhance reprogramming. We noticed earlier studies such as reported during chronic biliary cirrhosis progression that p38 seems to sustain hepatocyte proliferation, and its deficiency leads to an increased proportion of binucleated hepatocytes [29]. On the other hand, it has been reported that p38/MAPK signaling could drive WGD [30]. Based on these seemingly contradictory findings, we decided to test if p38 plays any role in WGD we observed in MEFs. To test this idea, we treated MEFs with SB203580, a widely used inhibitor of p38 MAPK kinase [30, 31], and show, surprisingly, that it actually reduces the proportion of BNCs (Fig. S5A–B). By live-cell imaging FUCCI system, we show that this p38i inhibits the generation of BNCs by preventing CF (Fig. 3A–B, Video S9–10). Consistently, we show with PI staining for DNA content that it reduces 8 N cells significantly (Fig. S5C–D). These results suggest that p38 promotes CF responsible for the observed WGD in our system.

We therefore postulate that p38i may facilitate iPSCs generation by removing the WGD-imposed barrier to reprogramming. To test this, we added p38i SB20580 to medium during different periods of the reprogramming experiments (Fig. 3C). Our findings demonstrate that p38i enhances reprogramming efficiency only when introduced during the pre-treatment phase of MEFs culture. Notably, continuous SB203580 treatment during the entire induction period (days 0–7) results in near-complete inhibition of reprogramming (Fig. 3D–E), consistent with previous reports on the role of p38 during reprogramming [32, 33].

To understand the phase-specific effect of p38i, we analyzed the transcriptional changes during reprogramming under the three conditions, i.e., pre-p38i and p38i versus DMSO, by harvesting cells at various time points (day 1, day 3, day 5, day 7), along with MEFs and ESCs or iPSCs as controls, for RNA sequencing and RT-qPCR. To further evaluate their kinetics from MEF to iPSCs under these conditions, we performed principal component analysis (PCA) that map trajectories of reprogramming and show that pre-p38i cells have a faster kinetics compared to the DMSO control, but p38i cells diverge from the expected course (Fig. 3F). In contrast to p38i, which suppresses the expression of pluripotent markers critical for cell programming, including Nanog, endogenous Oct4, and Dppa3, pre-p38i upregulates these core factors (Fig. 3G). These results demonstrate the differential impact of p38i at different stages of reprogramming and confirm the primarily promoting phase for p38i at the pre-induction period (See Fig. 3C).

In contrast to the pre-induction period, p38i has a totally different effect to inhibit reprogramming completely during induction. To understand this difference, we decided to examine the impact of p38i on a critical gateway to reprogramming, i.e., Mesenchymal-epithelial transition (MET) which we and others reported as an initiating event for reprogramming [18, 19, 34]. As expected, p38i when applied to the induction phase, renders MEFs unable to undergo typical epithelial-like transition as revealed by immunofluorescence analysis of markers such as CDH1 and NANOG. Compared to the control, p38i blocks the up regulation of epithelial-associated genes (Cdh1, Krt8, Cldn3), while mesenchymal genes (Twist1, Zeb1) exhibit no significant downregulation (Fig. S6A-D). Together, these results demonstrate that the inhibition of p38 in pre-induction phase prevents WGD in MEFs and facilitates iPSC reprogramming, but blocks reprogramming by preventing MET at the induction phase.

### p53 is activated during WGD to block reprogramming

**Fig. 4:**
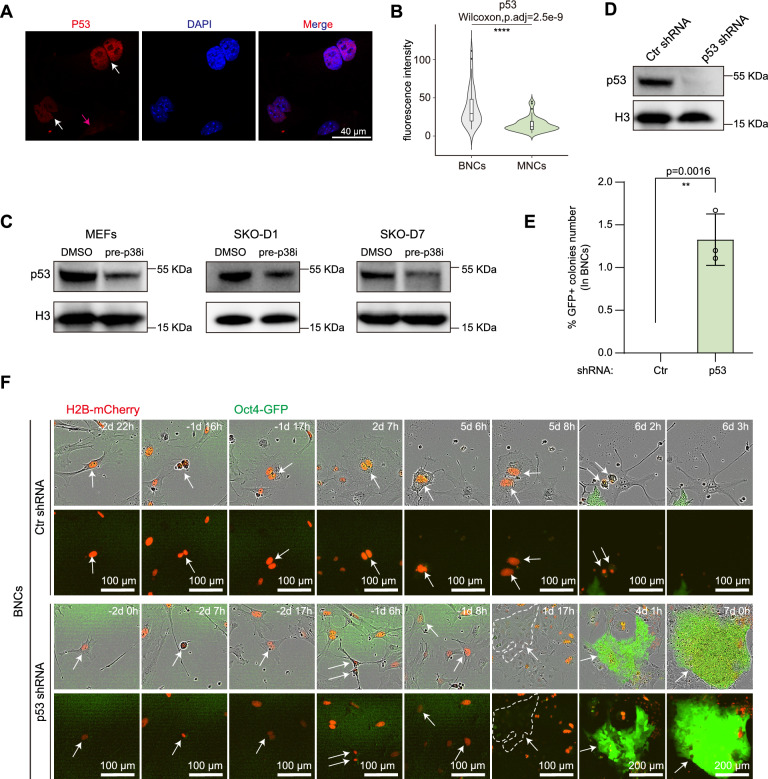
p53 prevents binucleated MEFs from reprogramming. **A**, Representative images of immunofluorescence for p53 in P3. The white arrow indicates BNCs, while the magenta arrow designates MNCs. Scale bars, 40 μm. **B**, The violin plots show the quantitative analysis of p53 fluorescence intensity in binucleated and mononucleated MEFs. More than 45 cells were analyzed in each group. Two-tailed Wilcoxon test adjusted for multiple comparisons. **C**, Immunoblots show the protein level of p53 in MEFs or at the indicated timepoints during reprogramming procedure. **D**, Western blot demonstrates specificity of our shRNA vectors. Lysates were extracted at D1 during cell reprogramming. **E**, Histogram presents the proportion of GFP + colonies number in BNCs from long term live-cell imaging during reprogramming when p53 knockdown and relative control. Total 200 BNCs were counted in 3 biological replicates and each biological replicate involved 8 views. Data are mean ± s.d., two-tailed, unpaired *t* test; **p < 0.01. **F**, Time course live-cell images of MEFs from mononuclear to binuclear during infected window and their cellular reprogramming procedure when p53 knockdown and relative control (see also video S11–S12). H2B-mCherry marks nucleus. Scale bars are as indicated. The arrows indicated the tracked cells and their daughter cells

We next wish to test the idea that novel barrier(s) activated during WGD prevent the reprogramming of MEFs. To probe the likely barriers, we turn to a known candidate, p53 as it is known to be activated in tetraploid cells as previously described [28] and the p53 pathway also acts as a barrier to somatic cell reprogramming [35–38]. To this end, we show by immunofluorescence analysis that BNCs exhibit significantly elevated γH2AX puncta and p53 protein levels compared to MNCs (Fig. S7A, B, Fig. 4A-B). Thus, these results suggest that DNA damage may be a major contributor to p53 activation in WGD, which is consistent with previous studies[7, 39]. Consistently, we also show MEFs treated with p38i appears to have less p53 expression (Fig. S8A-B). These results indicate p53 is activated in MEFs having underwent WGD. Indeed, we show that, with western blotting experiments to assess p53 protein expression at various timepoints during programming, there are significant reductions in p53 protein levels both in MEFs and at defined reprogramming timepoints (day 1 and day 7) with p38i pretreatment (Fig. 4C). These results suggest that p38i pretreatment enhances reprogramming efficiency by suppressing CF-induced WGD through p53 reduction.

To establish a causal relationship between p53 and capability of reprogramming in WGD MEFs, we performed p53 knockdown experiments during reprogramming (Fig. 4D) and live-cell imaging to trace BNCs formed during viral infection and assessed their potential to generate GFP + colonies. Our quantitative imaging analysis reveal that p53-depleted BNCs regain the ability to reprogram to form GFP + colonies (Fig. 4E–F, Video S11–12). Based on above findings, we can establish 4N-iPSC cell lines by knockdown p53 during cell reprogramming in BNCs with live-cell trace to pick up WGD GFP + colonies (Fig. S8C–D). The expression of canonical pluripotent markers (Rex1, Dppa5a, Nanog, Esrrb) shows no significant different between 2N-iPSCs and 4N-iPSCs (Fig. S8E). Together, these results demonstrate that p53 is activated during WGD and the resulting BNCs become resistant to reprogramming.

## Discussion

**Fig. 5:**
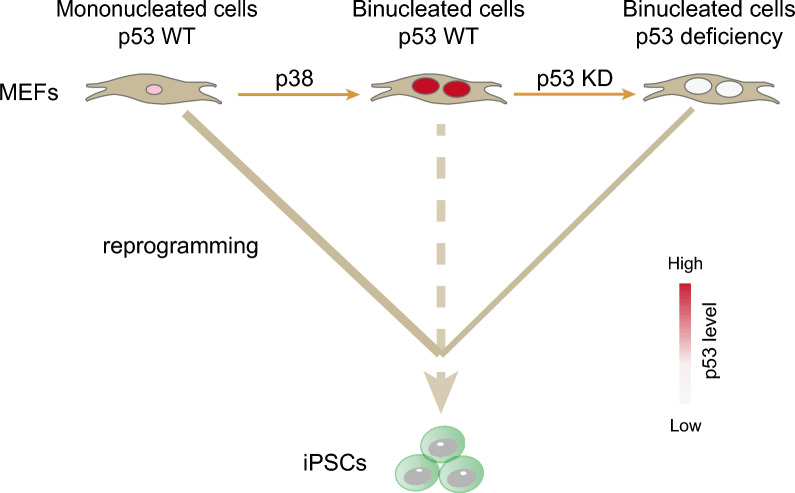
A model for WGD-mediated restriction of somatic cell reprogramming. *In vitro* cultured MEFs undergo spontaneous WGD and the resulting tetraploid cells are refractory to iPSCs reprogramming. p38 serves as the driver of cytokinesis failure induced WGD and the consequent accumulation of p53 in these binucleated MEFs acts as the intrinsic barrier for iPSCs reprogramming

We show here that MEFs undergo spontaneous WGD, mainly via cytokinesis failure or CF, and the resulting tetraploid cells are refractory to iPSC reprogramming. Our findings reveal p38 acts as a driver of WGD, while p53 serves as a brake on cell fate transition following WGD (Fig. 5). Our findings reveal that spontaneous genome duplication in cultured MEFs is p38-dependent, suggesting these cells may exist in a stress-activated state following isolation. While the precise nature of these stimuli and the upstream signaling mechanisms activating p38 remain to be elucidated, these observations may inspire further investigations into questions associated with WGD in general and the biology of tetraploid cells in particular.

As a pivotal node in the MAPK signaling pathway, p38 regulates numerous downstream substrates and influences diverse biological processes in a context-dependent manner. In primary MEFs culture conditions, activated p38 inhibits cell cycle kinases, causing mitotic defects in MEFs [30]. During somatic cell reprogramming, however, Yamanaka factors alleviate this suppression and promote cell cycle progression [40], potentially counteracting p38 inhibitory effect. Additionally, p38 facilitates MET, contributing positively to the reprogramming process. Among the four p38 isoforms, p38α is the predominant one expressed in MEFs and during reprogramming, suggesting it may paly a central role in these processes.

Polyploidy is commonly observed in plants, fish and amphibians [41, 42]. Compared to diploid counterparts, polyploid offspring exhibit novel advantageous traits such as larger body size, accelerated growth rate, and enhanced reproductive capacity [43]. Polyploid breeding has been widely employed in crop improvement programs. However, this phenomenon remains exceptionally rare in higher vertebrates, particularly birds and mammals. The underlying mechanisms why higher vertebrates are so resistant to whole genome duplication continue to pose an unresolved scientific question. Our study could provide critical insights for establishing WGD-iPSCs in other species, offering new perspectives to investigate tetraploid biology in higher vertebrates and opening new avenues for utilizing polyploidy in sustainable animal breeding strategies.

We show that binucleation in MEFs occurs readily *in vitro*, although it is rarely observed during development *in vivo*, which is likely due to the insufficient culture conditions to sustain long-term genomic stability in primary cells after isolation. Nevertheless, the tetraploid cell model remains biologically relevant for studying cell fate transition, as growing evidence indicates that tetraploidization takes place in diverse somatic cell types *in vivo*, like cardiomyocytes, hepatocytes and epithelial cells during processes including development, aging and tumorigenesis [44–47]. This phenomenon is frequently associated with subsequent p53 activation and cell cycle arrest, pointing to the consistency surveillance mechanism. Thus, we suppose that binucleation in MEFs culture may serve as an *in vitro* sensitive model for elucidating the molecular mechanism of WGD and its downstream effects.

Other strong interest in WGD is related to tumorigenesis. To this end, we have show that p53 is a barrier to cell fate transition in tetraploid cells as shRNAs for p53 relieves the barrier to generate tetraploid iPSCs. This may be relevant to tumorigenesis as spontaneous WGD acquires higher levels of p53 that will safeguard these unscheduled tetraploid cells from tumorigenesis, i.e., further cell fate transitions as we show with our reprogramming assays. These results further suggest that WGD does not automatically endow the resulting tetraploid cells to undergo tumorigenesis which only occurs when the barrier is lower or removed as we demonstrated with shRNAs against p53 during reprogramming. In a sense, the experimental systems we provided here may be a model to further analyse the relationship between WGD and tumorigenesis experimentally. Meanwhile, this spontaneous WGD system may be studied without the need for cytotoxic chemical inhibitors typically employed to induce polyploidization.

## Materials and methods

###  Animals

Oct4-GFP (OG2) reporter-allele-carrying mice (CBA/CaJ x C57BL/6 J) were original from The Jackson Laboratory (Mouse strain datasheet: 004654). The wild-type female 129 mice were purchased from Vital River Laboratory Animal Technology Co., Ltd (Beijing). All mice were housed in a temperature-controlled room about 20–26 °C with a 12 h light/dark cycle, whose humidity is 40–70%. The animal studies were performed according to the applicable guidelines and regulations of the Institutional Animal Care and Use Committee of Westlake University (Animal Protocol No. 23-109-PDQ-7), Hangzhou, China.

### Cell lines and cell culture

Plat-E cells were maintained in DMEM/high-glucose (Hyclone, SH30022-2B) supplemented with 15% FBS (Vazyme, F103-01), 1% GlutaMAX (GIBCO, 35,050,061) and 1% NEAA (GIBCO, 11,140,035), named as 15% FBS medium. OG2 MEFs were isolated from E13.5 embryos (female or male) by crossing male OG2 mice to 129 female mice. In briefly, the embryos were isolated and cut into small pieces. Then the tissues were digested with digestive solution (0.25% trypsin: 0.05% trypsin = 1:1; GIBCO, 25200072, 25300054) for 15 min at 37 °C. The isolated cells were plated onto 0.1% gelatin (Millipore, ES-006-B) coated culture dish and cultured in 15% FBS medium. All of the cell lines have been confirmed as mycoplasma contamination free with the kit from Beyotime (C0298M).

###  Plasmids and molecular cloning

pMXs retroviral vectors expression mouse Sox2, Klf4, and Oct4 were purchased from Addgene. To construct the FUCCI reporter for cell cycle labeling, a FUCCI cassette containing Cdt1 (30–120) and Gem1 (1–110) tagged with two fluorescent proteins, flanked by T2A self-cleaving peptide elements, was derived from pBOB-EF1-FastFUCCI-Puro obtained from MiaoLingBio, China. The original fluorescent proteins were replaced with mCherry and mAzamiGreen (mAG), and then the modified cassette was transferred to the pMXs retroviral vector. The H2B reporter, which indicates cell nucleus, was constructed by fusing human H2B DNA sequence with mCherry and cloning them into the pMXs retroviral vector. pRetroSuper vectors containing shRNA sequences for p53 (GTACATGTGTAATAGCTCC) [25].

### iPSCs generation

Plat-E cells were transfected with plasmids using polyethylenimine reagent (PEI, Yeasen, MW40000) to produce retroviral supernatants. OG2-MEFs were plated onto 12-well plate at 3 × 104 cell density per well, and then infected with the retroviral supernatants. After two rounds of 24 h infection, which we designated as day 0, the medium was changed to iCD1 reprogramming induing medium. The fresh medium was changed daily until day 7. Oct4-GFP positive colonies were scanned by living cells station (Keyence, Japan), and counted using Image-J software (v1.54f, NIH).

###  Immunoblotting

Cells were harvested and lysed in lysis buffer supplemented with protease inhibitor cocktail (Roche) on ice for 15 min. Subsequently, the lysates were sonicated with Bioruptor Plus (Diagenode) and boiled at 100 °C for 10 min. After centrifugation, the cell supernatants were subjected to SDS–PAGE and transferred onto the PVDF membrane. After being blocked with 5% nonfat milk for 2 h at room temperature, the membranes were successively incubated with primary antibodies and then secondary antibodies. Finally, the bands were detected using SuperPico ECL Chemiluminescence Kit (Vazyme, E422-02). Primary antibodies used were: anti-P53 (CST, 2524S, 1:1000), anti-H3 (Abcam, ab1791, 1:2000).

###  Flow cytometry analysis

To analyze the DNA content of MEFs, cells were collected and washed with precooled PBS. Then, samples were fixed and stained with PI staining kit (Beyotime, C1052) according to manufacturer’s instructions. PI intensity for cell DNA Content analysis detection was measured using CytoFLEX LX Flow Cytometer (Beckman Coulter) cytometers in the Y610 channel. FlowJo 10.4 (BD Biosciences) software was used for data analysis.

###  Senescence-associated β-galactosidase staining and quantification

MEF cells seeded in 6-well plates, were fixed and stained using the Senescence β-Galactosidase Staining Kit (Beyotime, C0602) following the manufacturer’s instructions. Staining was performed overnight at 37 °C in the dark. Cells were observed and imaged under bright field using an Olympus IX73 inverted microscope. Positive cells were counted and divided by the total number of cells, which were stained with Hoechst 33,342 and captured under fluorescence mode, to calculate the percentage of positive cells. For etoposide-induced senescence, MEF cells were treated with 20 µM etoposide for 2 days, followed by sub-culturing at a 1:3 ratio before proceeding with further analysis.

### Measurement of ROS

Intracellular ROS levels were detected by dihydrorhodamine 123 (MCE, HY-101894). Cells were cultured in 8-well chamber (Cellvis, C8-1.5H-N). During staining procedure, the cells were gently washed twice with PBS, followed by incubation with 10 μM dihydrorhodamine 123 and 10 μM Hoechst 33342 at 37 °C for 20 min, and then the cells were washed thrice with DMEM. Finally, imaging with a laser confocal microscope (ZEISS, LSM 980).

###  Immunofluorescence

Cells growing on coverslips were washed 2 times with PBS, then fixed with 4% PFA for 30 min, and subsequently penetrated and blocked with 0.1% Triton X-100 and 3% BSA for 30 min at room temperature. Then, the cells were incubated with primary antibody for overnight. After 4 washes in PBS, Sample was incubated with secondary antibodies for 1 h. After 5 washes for 5 min per time, cells were then incubated in DAPI (Sigma-Aldrich D9542) for 2 min. Then, the coverslips were mounted on the slides for observation on the confocal microscope (ZEISS, LSM 980). The following antibodies were used in this project: anti-P53 (CST, 2524S, 1:200), anti-HSP90 (HuaBio, ET1605-56, 1:500), anti-γH2AX (abcam, ab26350, 1:200). Expression was quantified average nuclear intensity with ZEN 2.6 lite software (ZEISS).

###  Karyotype analysis

Before karyotype analysis, MEFs were treated with 4 μg/mL colchicine in culture medium for 2.5 h. The cells were digested, centrifuged, resuspended with preheated 75 mM KCl hypotonic solution, and incubated at 37 °C for 20 min. After that, MEFs were fixed with Carnoy’s solution (3:1 (v/v) mix of methanol and acetic acid) for 30 min at room temperature, and then centrifuged, resuspended with Carnoy's solution. Finally, the MEFs were dropped on precooled slides, dried, stained with Giemsa, photoed and arrange karyotype by Guangzhou Haoyu Biotechnology Co., Ltd.

###  Live-cell imaging

MEFs stably expressing FUCCI reporter were plated about 12 h prior to imaging in a 6-well (corning,3615) dishes at original density of 4X10^4^–5X10^4^ cells per well. Time-lapse live-cell imaging was performed using Incucyte® SX5 microscope that maintains temperature at 37 °C and CO_2_ at 5%. Phase-contrast and fluorescent images were taken every 20 min with a 10 × objective. long-term live-cell imaging during cell reprogramming was conducted as illustrated (Fig. 2A). Filter sets and exposure times were default that no phototoxicity or photobleaching was observed in cells. Medium was changed every day. Image processing was performed using its software.

###  RNA extraction and quantitative real-time (qRT)-PCR and RNA-seq

Total RNA was extracted using the FastPure Cell/Tissue Total RNA Isolation Kit V2 (Vazyme, RC112-01) following the manufacturer’s protocol and quantified with a Multiskan SkyHigh Microplate Spectrophotometer (Thermo Fisher Scientific). The extracted RNA was then reverse transcribed using HiScript II Q RT SuperMix for qPCR (Vazyme, R222-01). qRT-PCR was performed using ChamQ SYBR Color qPCR Master Mix (Vazyme, Q411-03) on a CFX96 Touch Real-Time PCR Detection System (Bio-Rad). All experiments were conducted in triplicate, and the primer sequences are provided in the Supplementary Table 1. For RNA-seq, VAHTS mRNA-seq V3 Library Prep Kit for Illumina (NR611, Vazyme) was used for library constructions and sequencing done with NextSeq500 Mid output 150 cycles (FC-404–2001, Illumina).

## Statistical information

Data are presented as mean ± s.d. as indicated in the figure legends. Unpaired two-tailed student *t* test, The *P* value was calculated with the Prism 6 software. A *p*-value < 0.05 was considered as statistically, *p < 0.05, **p < 0.01, ***p < 0.001, ****p < 0.0001. No statistical method was used to predetermine sample size. The experiments were not randomized. The investigators were not blinded to allocation during experiment and outcome assessment.

## Supplementary Information

Additional file1

Additional file2

Additional file3

Additional file4

Additional file5

Additional file6

Additional file7

Additional file8

Additional file9

Additional file10

Additional file11

Additional file12

Additional file13

Additional file14

## Data Availability

The RNA-seq data have been deposited in the Gene Expression Omnibus database under the accession code GSE295779.

## Abbreviations

MEFs: Mouse embryonic fibroblasts
WGD: Whole-Genome Duplication
iPSCs: Induced pluripotent stem cells
MET: Mesenchymal-to-epithelial transition
CF: Cytokinesis failure
MS: Mitotic slippage
EnR: Endoreplication
BNCs: Binucleated cells
MNCs: Mononucleated cells
DCB: Dihydrocytochalasin B
