# Supplementary material for: Spontaneous whole genome duplication renders mouse embryonic fibroblasts resistant to reprogramming
Source: Cell Biosci. 2026 Mar 26;16:51. doi: 10.1186/s13578-026-01558-3 (PMC13147676; doi:10.1186/s13578-026-01558-3)
Supplement: Supplementary file 1 — Additional file1 [file 13578_2026_1558_MOESM1_ESM.pdf]

Figure S1

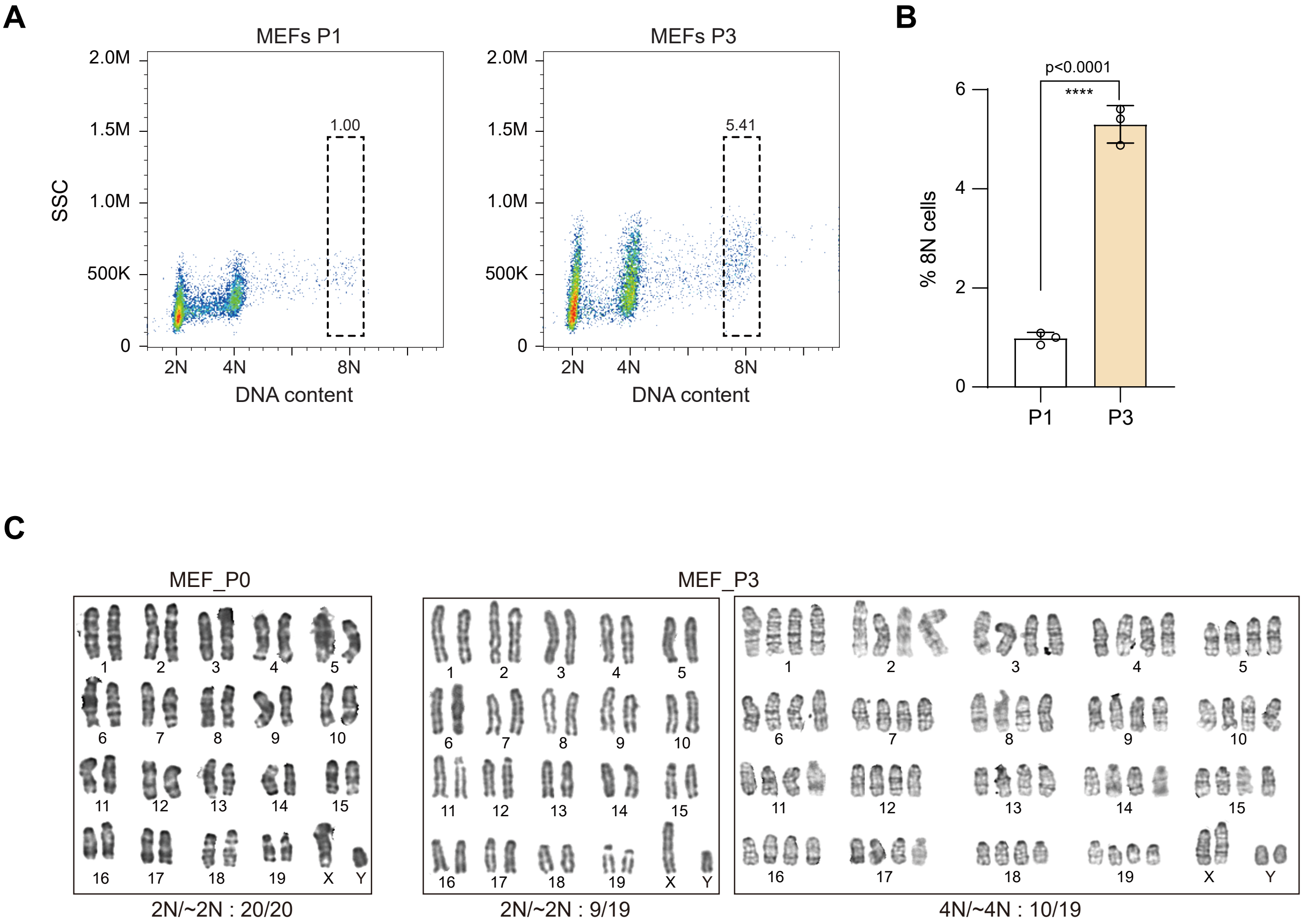

A
MEFs P1
MEFs P3
2.0M
1.5M
1.0M
500K
0
SSC
1.00
5.41
2N
4N
8N
DNA content
B
p<0.0001
****
% 8N cells
6
4
2
0
P1
P3
C
MEF_P0
MEF_P3
1 2 3 4 5
6 7 8 9 10
11 12 13 14 15
16 17 18 19 X Y
2N/~2N : 20/20
2N/~2N : 9/19
4N/~4N : 10/19

Figure S1 Tetraploid cells are detected in primary MEFs in vitro. A, DNA contents are analyzed by Propidium Iodide (PI) via flow cytometry in MEFs of P1 and P3. B, Histogram shows the proportions of 8N cells in MEFs of P1 and P3. Data are mean±s.d., two-tailed, unpaired t-test, three independent experiments. ****$p < 0.0001$. C, The representative karyotype for MEFs of P0 and P3.

Figure S2

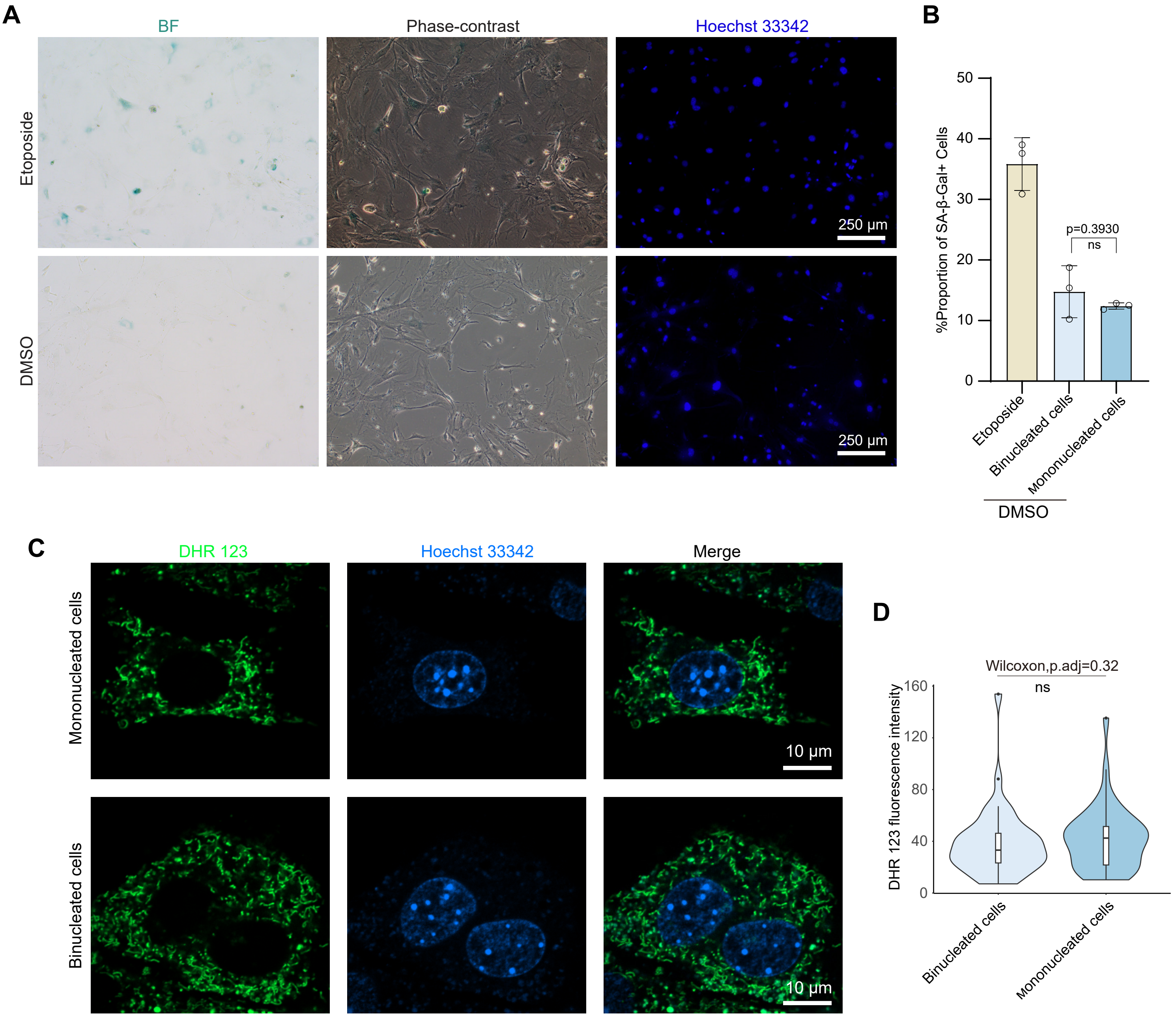
A
BF
Phase-contrast
Hoechst 33342
Etoposide
DMSO
250 μm
250 μm
B
%Proportion of SA-β-Gal+ Cells
50
40
30
20
10
0
p=0.3930
ns
Etoposide
Binucleated cells
Mononucleated cells
DMSO
C
DHR 123
Hoechst 33342
Merge
Mononucleated cells
Binucleated cells
10 μm
10 μm
D
Wilcoxon,p.adj=0.32
ns
DHR 123 fluorescence intensity
160
120
80
40
0
Binucleated cells
Mononucleated cells

Figure S2 The effect of cell stress on the WGD in MEFs. A, Typical images of the β-gal staining in mononucleated and binucleated cells of MEFs. Scale bars, 250μm. B, Histogram displays the proportion of SA-β-gal^{+} cells in mononucleated and binucleated cells. Data are mean ± s.d., two-tailed, unpaired t test; cell were counted in 3 biological replicates and each biological replicate involved one view, ns, no significance. C, Representative images of ROS staining with 10 μM Dihydrorhodamine 123 (DHR 123) (MCE, HY-101894) fluorescence probe in mononucleated and binucleated cells of P3 MEFs. Scale bars, 10μm. D, The violin plots show the quantitative analysis of DHR 123 fluorescence intensity in binucleated and mononucleated MEFs. Total 40 cells were analyzed in each group. Two-tailed Wilcoxon test adjusted for multiple comparisons, ns, no significance.

Figure S3

A

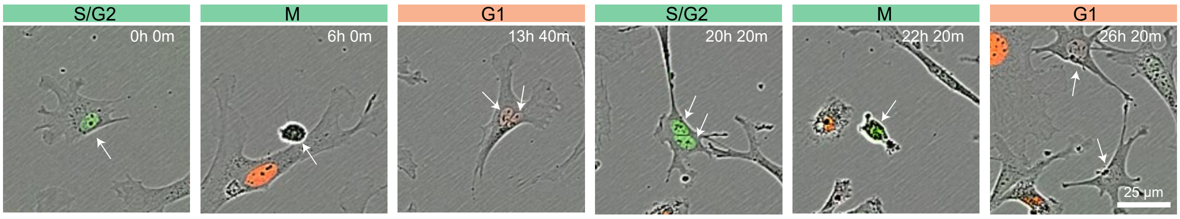

Figure S3 Living-cell tracking of the transition from binucleated cells to mononucleated tetraploid cells. A, Time-lapse images showing transition binucleated cells to mononucleated tetraploid cells in P3 MEFs. Scale bars, 25μm. The white arrows indicated the tracked cells and their daughter cells.

Figure S4

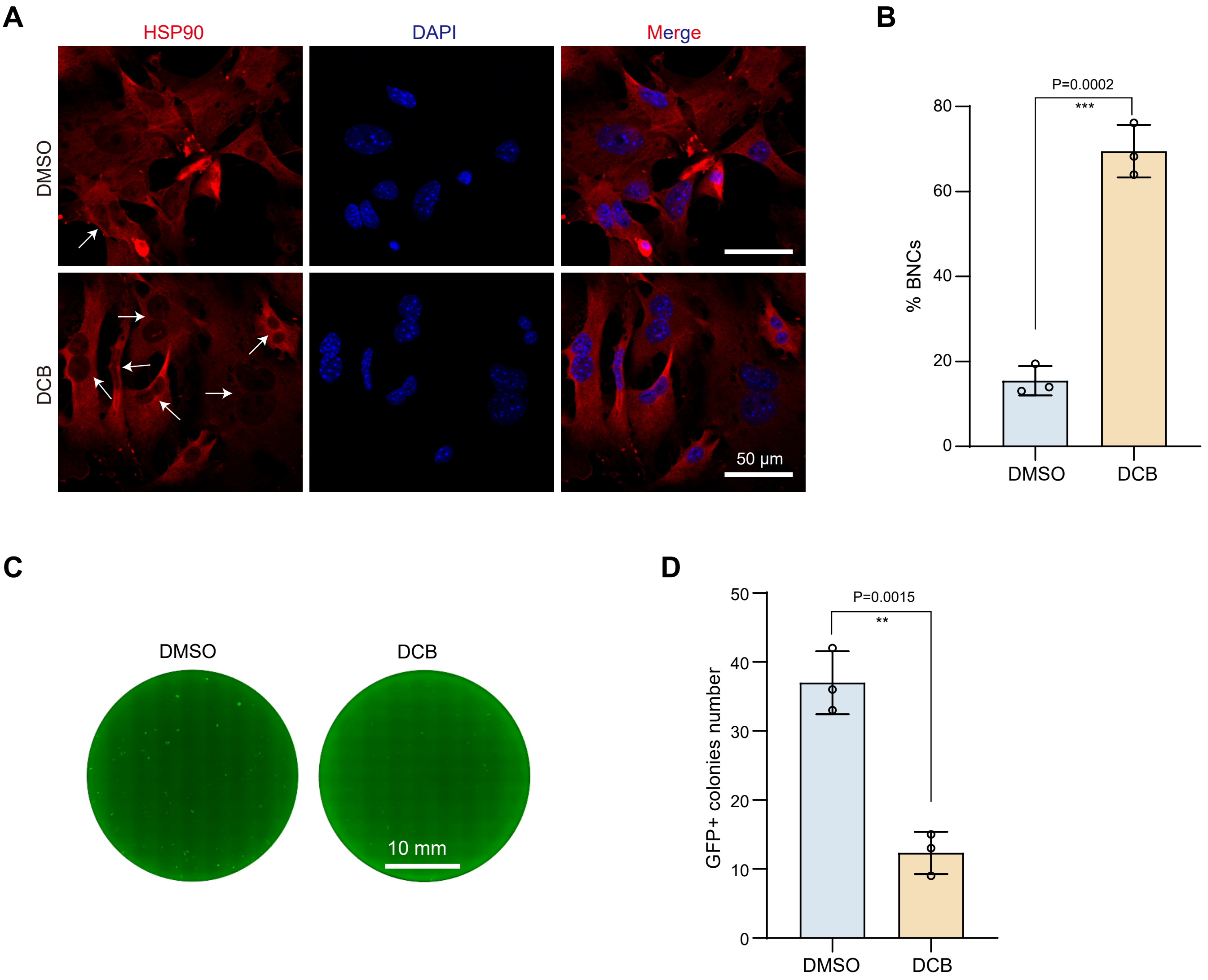
A
HSP90
DAPI
Merge
DMSO
DCB
50 μm
B
P=0.0002
***
% BNCs
80
60
40
20
0
DMSO
DCB
C
DMSO
DCB
10 mm
D
P=0.0015
**
GFP+ colonies number
50
40
30
20
10
0
DMSO
DCB

Figure S4 DCB-induced binucleated MEFs exhibit diminished reprogramming potential. A, Representative images of immunofluorescence for HSP90 in P3. The white arrow indicates BNCs. Scale bars, 50μm. B, Histogram displays the proportions of BNCs with or without DCB. Data are mean±s.d., two-tailed, unpaired t-test, N = 3 strains. ***$p < 0.001$. C, Representative images of GFP+ colonies at D7. D, Histogram shows the GFP+ colonies number at D7 in indicated group. Data are mean ± s.d., two-tailed, unpaired t test; n = 3 independent experiments, **$p < 0.01$.

Figure S5

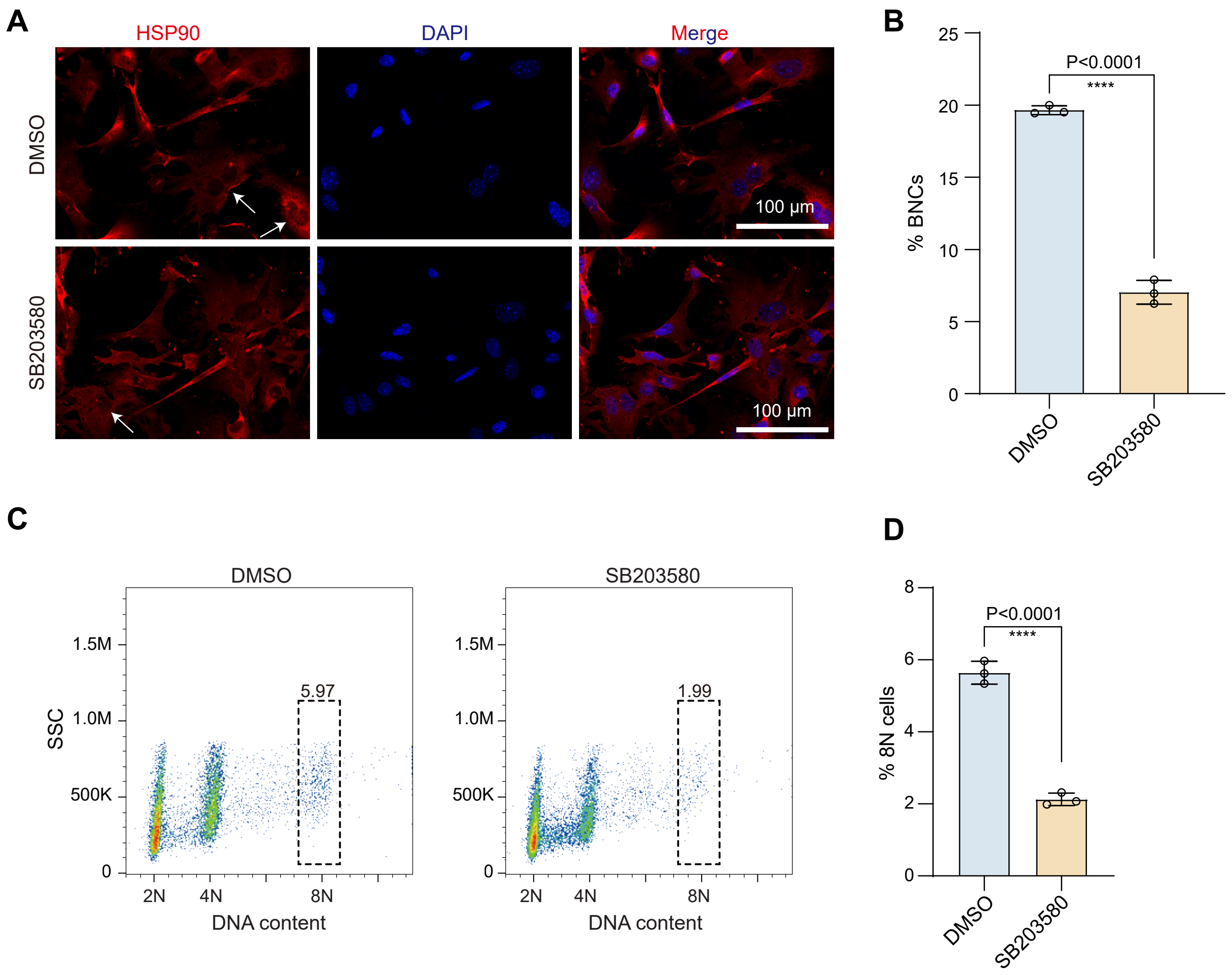
A
HSP90
DAPI
Merge
DMSO
SB203580
100 µm
100 µm
B
% BNCs
P<0.0001
****
25
20
15
10
5
0
DMSO
SB203580
C
DMSO
SB203580
SSC
1.5M
1.0M
500K
0
5.97
1.99
2N
4N
8N
DNA content
D
% 8N cells
P<0.0001
****
8
6
4
2
0
DMSO
SB203580

Figure S5 p38 MAPK inhibitor SB203580 reduces BNCs in primary MEFs. A, Representative images of immunofluorescence for HSP90 with or without treatment of SB203580. The white arrow indicates BNCs. Scale bars, 100μm. B, Histogram displays the proportions of BNCs with or without treatment of SB203580. Data are mean±s.d., two-tailed, unpaired t-test, N = 3 strains. ****$p < 0.0001$. C, DNA contents are analyzed by Propidium Iodide (PI) via flow cytometry in MEFs of P3 with or without treatment of SB203580. D, Histogram shows the proportions of 8N cells in MEFs of P3 with or without treatment of SB203580. Data are mean±s.d., two-tailed, unpaired t-test, three independent experiments. ****$p < 0.0001$.

Figure S6

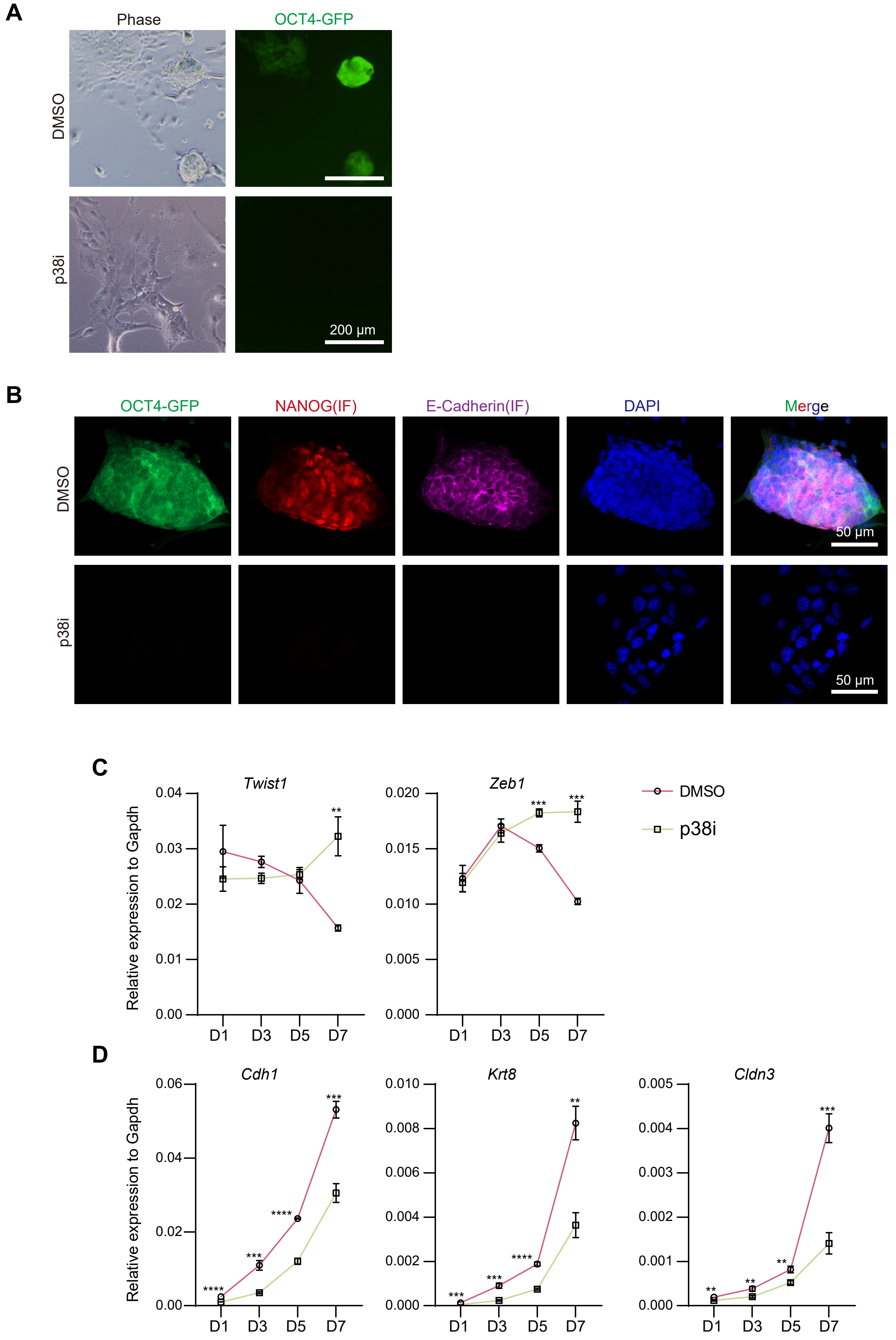

A
Phase
OCT4-GFP
DMSO
p38i
200 μm
B
OCT4-GFP
NANOG(IF)
E-Cadherin(IF)
DAPI
Merge
DMSO
p38i
50 μm
50 μm
C
Twist1
Zeb1
Relative expression to Gapdh
0.04
0.03
0.02
0.01
0.00
0.020
0.015
0.010
0.005
0.000
D1
D3
D5
D7
DMSO
p38i
D
Cdh1
Krt8
Cldn3
Relative expression to Gapdh
0.06
0.04
0.02
0.00
0.010
0.008
0.006
0.004
0.002
0.000
0.005
0.004
0.003
0.002
0.001
0.000
D1
D3
D5
D7

Figure S6 Inhibition of p38 MAPK during cellular reprogramming period impedes generation of mouse iPSCs. A, Representative cell morphological micrograph during cellular reprogramming at D7 in iCD1 medium with or without SB203580. Scale bars, 200μm. B, Expression patterns of pluripotency markers, Oct4 (ectopic viral expression), Nanog and E-Cadherin during cellular reprogramming at D7 in iCD1 medium with or without SB203580. Scale bars, 100μm. C-D, Expression of epithelial genes (Cdh1, Krt8, Cldn3) (C) and mesenchymal genes (Twist1, Zeb1) (D) were determined by qPCR on days 1, 3, 5 and 7 during reprogramming. Data are mean ± s.d., two-tailed, unpaired t test; n = 3 biological replicates. **p<0.01; ***p<0.001; ****P<0.0001.

Figure S7

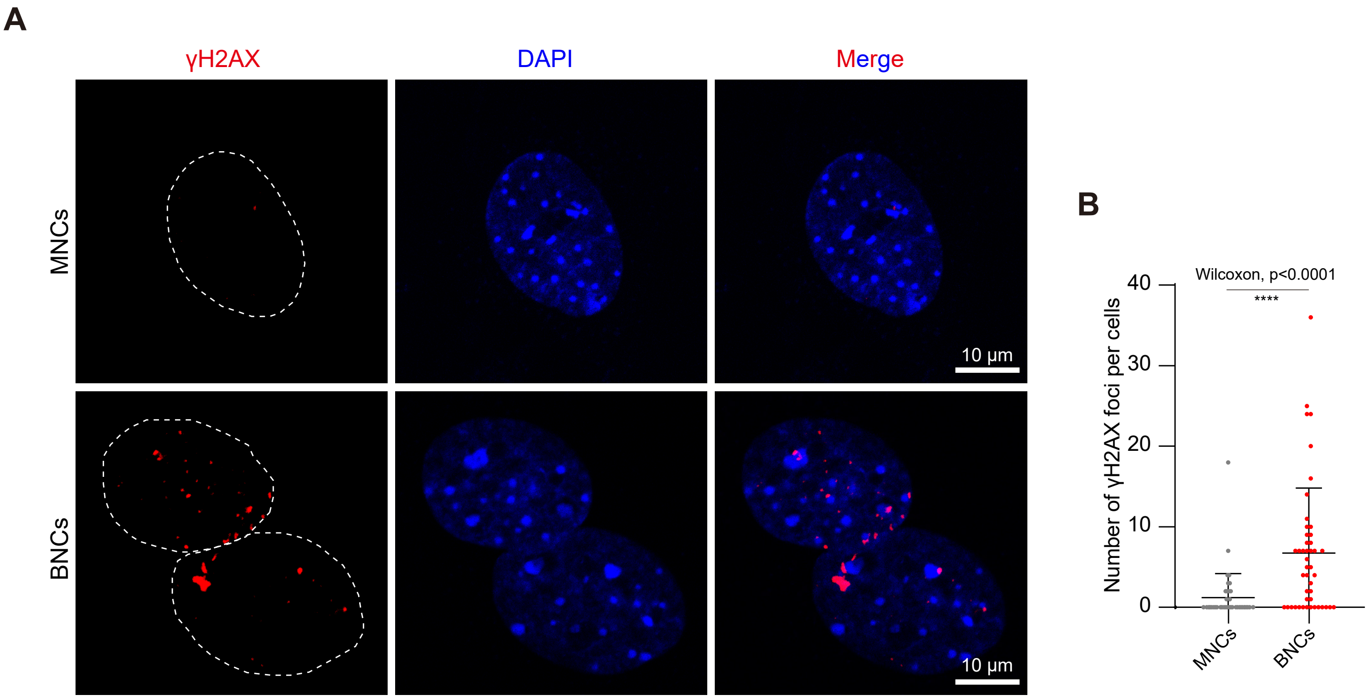
A
γH2AX
DAPI
Merge
MNCs
BNCs
10 µm
10 µm
B
Wilcoxon, p<0.0001
****
Number of γH2AX foci per cells
40
30
20
10
0
MNCs
BNCs

Figure S7 The DNA damage in WGD MEFs. A, Representative images of immunofluorescence for γH2AX in MNCs and BNCs of MEFs. Scale bars, 10μm. B, The number of γH2AX foci per cells in MNCs and BNCs of MEFs. About 45 cells were analyzed in each group. Two-tailed Wilcoxon test adjusted for multiple comparisons.

Figure S8

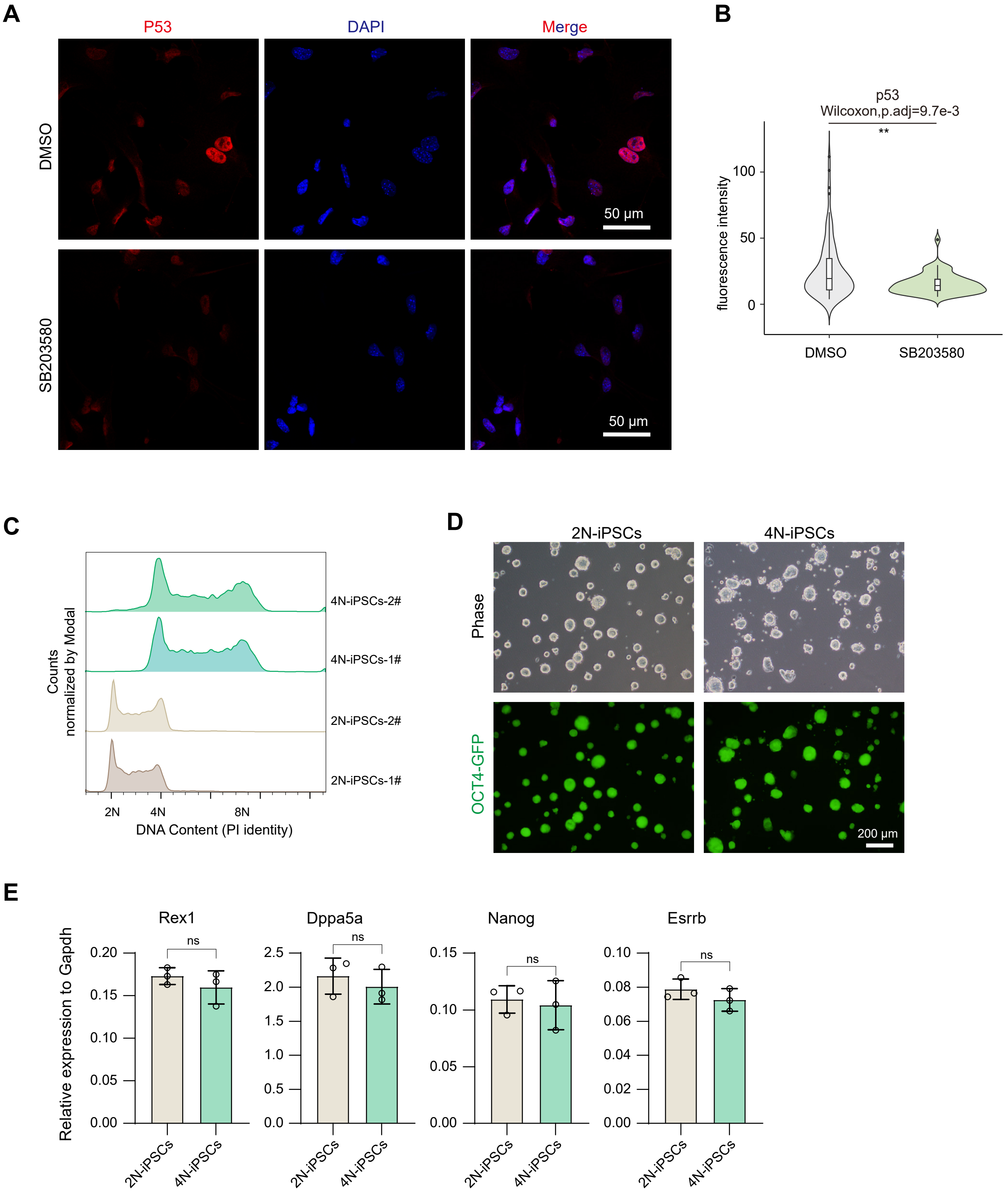

A
P53
DAPI
Merge
DMSO
SB203580
50 μm
50 μm
B
p53
Wilcoxon,p.adj=9.7e-3
**
fluorescence intensity
100
50
0
DMSO
SB203580
C
Counts
normalized by Modal
4N-iPSCs-2#
4N-iPSCs-1#
2N-iPSCs-2#
2N-iPSCs-1#
2N
4N
8N
DNA Content (PI identity)
D
2N-iPSCs
4N-iPSCs
Phase
OCT4-GFP
200 μm
E
Relative expression to Gapdh
Rex1
Dppa5a
Nanog
Esrrb
ns
ns
ns
ns
0.20
0.15
0.10
0.05
0.00
2.5
2.0
1.5
1.0
0.5
0.0
0.15
0.10
0.05
0.00
0.10
0.08
0.06
0.04
0.02
0.00
2N-iPSCs
4N-iPSCs

Figure S8 Exposure to p38 MAPK inhibitor SB203580 suppresses p53 protein level in primary MEFs and establishment of 4N-iPSCs. A, Representative images of immunofluorescence for p53 in P3 MEFs, either in the presence or absence of SB203580. Scale bars, 50μm. B, The violin plots show the quantitative analysis of p53 fluorescence intensity, either in the presence or absence of SB203580. In the two designated groups, more than fifty cells were analyzed per group. Two-tailed Wilcoxon test adjusted for multiple comparisons. C, DNA contents are analyzed by Propidium Iodide (PI) via flow cytometry in 2N-iPSCs and 4N-iPSCs. D, Representative images of 2N-iPSCs and 4N-iPSCs. Scale bars, 200μm. E, RT–qPCR analysis of the expression of representative pluripotent genes in 2N-iPSCs and 4N-iPSCs. Data are mean ± s.d., two-tailed, unpaired t test; from 3 iPSC clones. ns, not significant.
